# Supplementary material for: The Association between Vision Impairment and Depression: A Systematic Review of Population-Based Studies
Source: J Clin Med. 2022 Apr 25;11(9):2412. doi: 10.3390/jcm11092412 (PMC9103717; doi:10.3390/jcm11092412)

Supplementary Materials

Supplementary Materials S1 - Full Search Strategy 2
Supplementary Materials S2 - Risk of Bias Assessment 3
Supplementary Materials S3 - Characteristics of included cross-sectional population-based studies 5
Supplementary Materials S4 - Quality assessment of included studies 7
Supplementary Materials S5 - Forest plot of overall prevalence of depression 8
Supplementary Materials S6 - Forest plot of overall prevalence of visual impairment 9
Supplementary Materials S7 - Forest plot of frequency of depression in visual impairment subjects ... 10
Supplementary Materials S8 - Forest plot of frequency of visual impairment in depressed subjects 11
Supplementary Materials S9 - Forest plot of unadjusted association between visual impairment and depression 12
Supplementary Materials S10 - Forest plot of adjusted association between vision impairment and depression 13
Supplementary Materials S11 - Forest plot of unadjusted association between VI and depression by depression diagnostic tool 14
Supplementary Materials S12 - Forest plot of unadjusted association between VI and depression by age inclusion criteria 15

Supplementary Materials S1 - Full Search Strategy

PUBMED

("Depressive Disorder"[Mesh] OR "Depressive Disorder, Treatment-Resistant"[Mesh] OR "Bipolar Disorder"[Mesh] OR "Adjustment Disorders"[Mesh] OR "Affective Disorders, Psychotic"[Mesh] OR depress*[TIAB] OR ((bipolar[TIAB] OR affective[TIAB] OR adjust*) AND disorder*[TIAB]))
AND
("Vision, Low"[Mesh] OR ((VISUAL[TIAB] OR vision[TIAB] OR ocular[TIAB] OR eye*[TIAB]) AND (IMPAIRMENT[TIAB] OR low[TIAB])))

EMBASE

('low vision'/exp OR ((visual:ab,ti OR vision:ab,ti OR ocular:ab,ti OR eye*:ab,ti) AND (impairment:ab,ti OR low*:ab,ti))) AND ('depression'/exp OR 'treatment resistant depression'/exp OR 'bipolar disorder'/exp OR 'adjustment disorder'/exp OR 'affective psychosis'/exp OR depress*:ab,ti OR ((bipolar:ab,ti OR affective:ab,ti OR adjust*:ab,ti) AND disorder*:ab,ti))
AND
('article'/it OR 'article in press'/it OR 'note'/it OR 'review'/it OR 'short survey'/it)
AND
[english]/lim

Supplementary Materials S2 - Risk of Bias Assessment

Details and operationalization of the risk of bias assessment
A maximum of 10 stars was assigned in five domains (max 2 for each domain).
Lack of generalizability bias
We assessed the lack of generalizability bias based on two criteria: sampling consistent with our study objectives and an available definition of visual loss used as criterion to be included (clinic-based, case only) or be diagnosed (population-based) as having low vision.
Regarding patient sampling and spectrum, we included (Question 1) consecutive patients attending low-vision rehabilitation services who are not affected by juvenile vision disorders; and subjects representative of the general adult or older population regardless of vision status (Question2, cross-sectional), who were not affected by clinical depression at baseline (Question 3, longitudinal).
We decided to assign 2 stars to studies that reported the inclusion of such patients and did not exclude patients with co-morbidities. If study participants were selected against strict and selective inclusion criteria, the study was considered to be affected by lack of generalizability bias and, consequently, assigned 1 or no stars.
We decided *a priori* to downgrade randomized controlled trials for lack of generalizability. We included case-control studies (for questions 2 and 3) only if they were nested in population-based studies.
Record bias
We assigned two stars to prospective studies and one star to retrospective and registry-based studies.
Attrition bias
This assessment differed according to study design and considered the proportion and the characteristics of patients excluded or lost to follow-up.
For cross-sectional studies recruiting in low vision services we considered that the failure to include more than 10% of eligible patients was of concern, as was the fact that risk factors for depression, such as comorbidities, were more prevalent in patients who were lost.
For cohort studies the assessment of attrition bias was based on the rate of loss to follow-up: we assigned two stars if the proportion of withdrawals was less than 5%; one star if it was greater than or equal to 5%, but less than 10%; and no stars if the rate was greater than or equal to 10%.
We assigned one or no stars to studies with a moderate or severe imbalance in patient characteristics concerning loss to follow-up and no stars to studies not reporting such data.
Detection bias
This assessment differed according to study design.
For cross-sectional studies we assigned 2 stars to studies using the same diagnostic modality for all subjects and regardless of vision status.
For longitudinal cohort studies we judged a period of observation of at least 24 months as optimal (2 stars) to assess detection bias; at least 12 months as intermediate (1 star); and less than 12 months as poor (zero stars). Moreover, we downgraded by 1 or 2 stars if the same diagnostic modality was not used for all subjects regardless of vision status.
We downgraded 1 star if a masked assessment with respect to vision status was not used or unclear.
Reporting bias
Our assessment of reporting bias focused on the pre-specification of methods used to diagnose depression and analyses methods.
We assigned 2 stars to studies reporting analyses for all pre-specified diagnostic tools, including psychiatric examination or validated questionnaires, for which a protocol was available.
We assigned 1 star to registry-based studies or studies reporting only a subset of pre-specified diagnostic tools.

No stars were assigned to studies reporting only the number of patients who experienced depression with no details on the diagnostic process.

Supplementary Materials S3 - Characteristics of included cross-sectional population-based studies

Study	Country	Data type	N°	Mean age or range	Ocular disease	Definition of visual impairment	Criteria for depression	Comorbidities
**Armstrong 2016 period 1**	United States	clinical examination	2,591	53	NS	SR	CESD-5	CDC Healthy Days Core (included)
**Armstrong 2016 period 2 2**	United States	clinical examination	3,599	58	NS	SR	CESD-5, PHQ-9	CDC Healthy Days Core (included)
**Bernabei, 2011**	Italy	survey	7,389	72	NS	SR	ICD-9	CaVD, CeVD
**Biddulph, 2014**	United Kingdom	administrative database	1,085	≥65	NS	NEI-VF	MHIS	HTN, CaVD, PD, DM, OAD
**Capella, 2005**	United States	survey	6,089	≥55	NS	SR	SR	HL
**Carabellese, 1993**	Italy	clinical examination	1,191	70-75	NS	< 20/50	BDI	HL
**Cheluvaraj 2015**	India	survey	254	>60	NS	<6/18	GDS-5	HTN, DM and refractive defects
**Cho, 2015**	Korea	survey	28,392	≥ 19	NS	<20/63	SR	DM, HTN
**Court, 2014**	United Kingdom	survey	291,169	≥65	NS	Read code	Read code	HTN, CaVD, Parkinson, MS, DM, epilepsy, stroke, HD, KD, PD, TD, others
**Crews, 2017**	United States	clinical examination	36,110	≥65	NS	SR	SR	HTN, CaVD, stroke, OAD, PD, cancer, DM, HD
**Evans, 2007**	United Kingdom	survey	13,900	81	AMD, cataract, refractive error, others	<6/18	GDS-15	CaVD, stroke, Parkinson, cancer, HL
**Garin, 2014**	Spain	survey	4,583	48	NS	SR	WHO-CIDI	CaVD, OAD, PD, DM, HTN, stroke
**Guthrie, 2016**	Canada, United States, Belgium, Finland	survey	550,360	≥65	NS	Clinical record	DRS	HL
**Hamedani, 2019**	United States	administrative database	47,582,342	≥65	NS	ICD-9	ICD-9	Anxiety, dementia, OAD
**Harada, 2008**	Japan	survey	644	≥65	NS	<0.5	GDS-5	HL, cancer, stroke, CaVD, DM
**Hirai, 2002**	United States	survey	484	50	DR	<20/40, severe <20/200	CES-D	CaVD, KD, limb amputation
**Karlsson 1998**	Iceland	survey	218	141 (18-69)- 77 (70-97)	Younger group: hereditary diseases Older group: AMD	20/60-20/200 Legal blindness <20/200	SR	NS
**Lee 2000**	Latinos in USA e Puerto Rico	survey	3,938 (391, 1514, 527)	20-74	NS	20/50	CES-D	NS
**Loprinzi 2013**	United States	clinical examination	567	60	NS	SR	PHQ-9	arthritis, CaVD, stroke, cancer, emphysema, chronic bronchitis, HTN
**Lupsakko, 2002**	Finland	survey	437	>75	NS	<20/60	DSM-IV	HL
**Lyness, 2006**	United States	clinical examination	546	75	NS	SR	DSM-IV	OAD, cancer, PD, DM, HL, CaVD

**Lyu, 2018a**	Korea	survey	2,167	≥45	NS	SR	CES-D	HTN, DM, cancer, CeVD, HD, CaVD, psychiatric disorder, OAD
**Lyu, 2018b**	Korea	survey	1,664	≥45	NS	SR	CES-D	HTN, DM, cancer, CeVD, HD, CaVD, psychiatric disorder, OAD
**Park, 2015**	Korea	administrative database	18,779	49	Glaucoma 34%, cataract 24.9%, eye and adnexa diseases 90.2%	ICD-10	ICD-10	CaVD, dementia, DM, PD, CTD, HD, KD, cancer, AIDS
**Rahman 2020**	Bangladesh	survey	400	50%>71	NS	SR	GDS-15	CaVD, stroke, DM, HTN
**Rovner, 1998**	United States	survey	872	76	NS	SR	Modified CES-D	NS
**Schuster, 2018**	Germany	survey	7,780	≥18	NS	SR	PHQ-9	NS
**Tsai, 2003**	Taiwan	survey	1,352	≥65	NS	<6/12	GDS-15	NS
**Van Nispen, 2015**	Netherland	clinical examination	1,237	≥55	NS	Severe 0.52-2 logMAR, mild 0.30-0.51 logMAR	CES-D	NS
**Wee, 2014**	Singapore	survey	559	≥60	NS	NS	GDS-15	HTN, CaVD, CeVD, OAD
**Zhang, 2013**	United States	clinical examination	10,480	≥20	NS	<20/40	PHQ-9	DM, CaVD, HTN, cancer

AMD= Age related Macular Degeneration; BDI= Beck Depression Inventory; CaVD= Cardiovascular Disease; CESDS= Center for Epidemiologic Studies Depression Scale; CeVD= Cerebrovascular Disease; CTD= Connective Tissue Disease; DM= Diabetes Mellitus; DRS= Depression Rating Scale; DR= Diabetic Retinopathy; DSM= Diagnostic and Statistical Manual of Mental Disorders; GDS= Geriatric Depression Scale; HD= Hepatic Disease; HL= Hearing loss; HTN= Hypertension; ICD= International Classification of Diseases; KD= Kidney Disease; OAD= Osteoarticular Disease; MD= Metabolic Disease; MHIS= Mental Health Inventory Screening; MS= Multiple Sclerosis; NEI-VF= National Eye Institute Visual Function; NS= Not specified; PD= Pulmonary disease; PHQ= Patient Health Questionnaire; SR= Self Report; TD= Thyroid Disease; VF= Visual Field; VI= Visual Impairment; WHO-CIDI= Word Health Organization Composite International Diagnostic Interview.

Supplementary Materials S4 - Quality assessment of included studies

Study	Year	Lack of generalizability bias	Record bias	Attrition bias	Detection bias	Reporting bias	Total score
**Armstrong**	2016a	**	**	**	**	*	9
**Armstrong**	2016b	**	**	**	**	*	9
**Bernabei**	2011	*	**	*	*	**	7
**Biddulph**	2014	*	**	*	*	**	7
**Capella**	2005	*	*	**	*	*	6
**Carabellese**	1993	**	**	**	*	**	9
**Cheluvaraj**	2016	**	**	**	**	*	9
**Cho**	2015	**	**	*	*	*	7
**Court**	2014	**	*	**	*		6
**Crews**	2017	*	*	**	*	*	6
**Evans**	2007	*	**	**	*	**	8
**Garin**	2014	*	**	*	*	**	7
**Guthrie**	2016	*	*	**	*	**	7
**Hamedani**	2019	**	*	**	*	**	8
**Harada**	2008	**	**	*	*	**	8
**Hirai**	2012	*	**	**	*	**	8
**Karlsson**	1998	*	**	*	**	*	7
**Lee**	2000a	**	*	**	**	*	8
**Lee**	2000b	**	*	**	**	*	8
**Loprinzi**	2013	*	*	**	**	**	8
**Lupsakko**	2002	*	**	*	*	**	7
**Lyness**	2006	*	**	**	*	**	8
**Lyu**	2018	*	*	*	*	**	6
**Park**	2015	*	*	**	*	**	7
**Rahman**	2020	**	**	**	**	**	10
**Rovner**	1998	*	**	**	*	**	8
**Schuster**	2018	*	*	**	*	**	7
**Tsai**	2003	*	**	*	*	**	7
**Van Nispen**	2015	*	*	*	*	**	6
**Wee**	2014	*	**	*	*	**	7
**Zhang**	2013	**	**	*	*	**	8

(*): 'star', so as each domain is assigned 0, 1 or 2 * (stars), with higher score meaning better quality

Supplementary Materials S5 - Forest plot of overall prevalence of depression

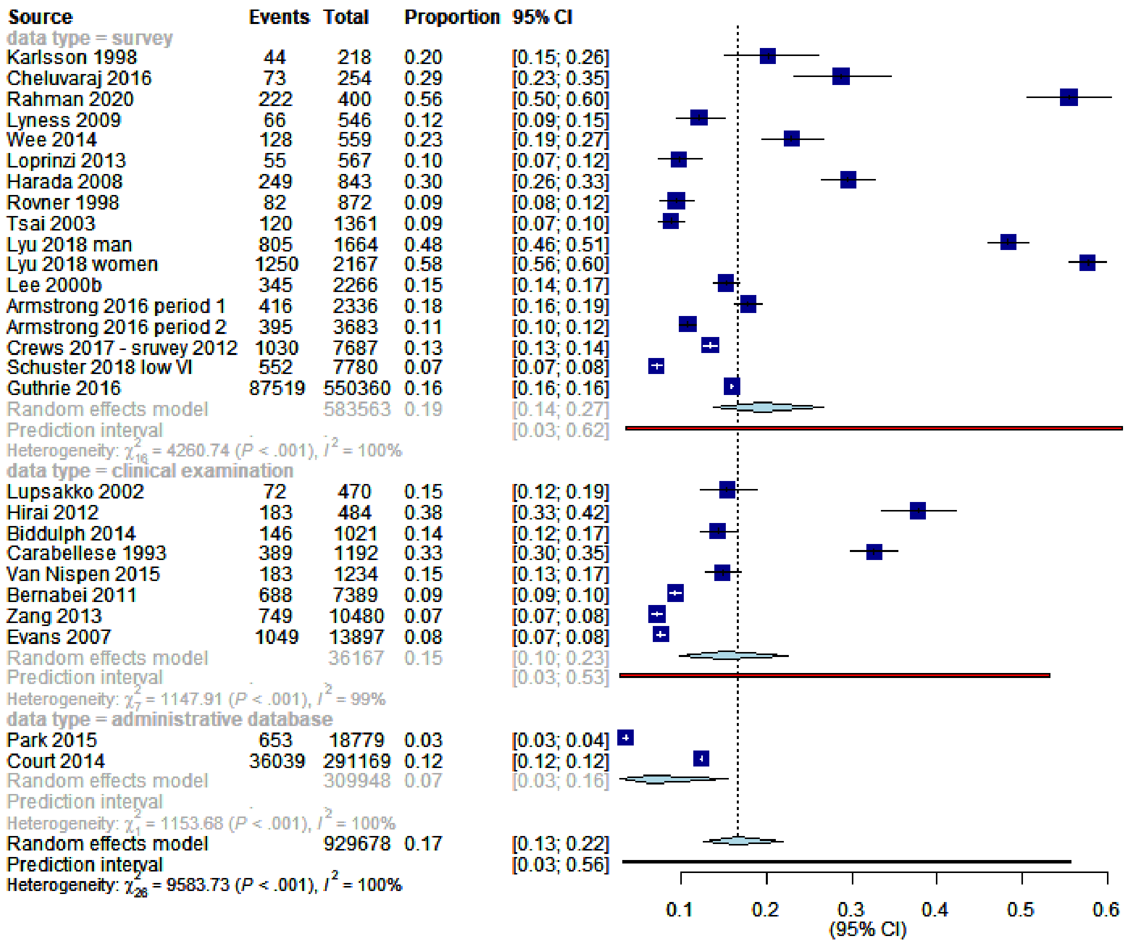

Supplementary Materials S6 - Forest plot of overall prevalence of visual impairment

Source	Events	Total	Proportion	95% CI
**data type = survey**				
Karlsson 1998	86	218	0.39	[0.33; 0.46]
Cheluvaraj 2016	121	254	0.48	[0.41; 0.54]
Rahman 2020	291	400	0.73	[0.68; 0.77]
Wee 2014	78	559	0.14	[0.11; 0.17]
Loprinzi 2013	47	567	0.08	[0.06; 0.11]
Harada 2008	243	843	0.29	[0.26; 0.32]
Rovner 1998	37	872	0.04	[0.03; 0.06]
Tsai 2003	98	1361	0.07	[0.06; 0.09]
Lyu 2018 man	348	1664	0.21	[0.19; 0.23]
Lyu 2018 women	648	2167	0.30	[0.28; 0.32]
Lee 2000b	95	2266	0.04	[0.03; 0.05]
Armstrong 2016 period 1	239	2336	0.10	[0.09; 0.12]
Armstrong 2016 period 2	350	3683	0.10	[0.09; 0.10]
Schuster 2018 low VI	708	7780	0.09	[0.08; 0.10]
Crews 2017 - survey 2012-2014	5258	36100	0.15	[0.14; 0.15]
Guthrie 2016	80634	550360	0.15	[0.15; 0.15]
Random effects model		611430	0.16	[0.10; 0.25]
Prediction interval				[0.02; 0.68]
Heterogeneity: χ^2_{15} = 2030.97 (P < .001), I^2 = 99%				
**data type = clinical examination**				
Lupsakko 2002	93	470	0.20	[0.16; 0.24]
Hirai 2012	27	484	0.06	[0.04; 0.08]
Biddulph 2014	178	1021	0.17	[0.15; 0.20]
Carabellese 1993	138	1192	0.12	[0.10; 0.14]
Van Nispen 2015	102	1234	0.08	[0.07; 0.10]
Bernabei 2011	104	7389	0.01	[0.01; 0.02]
Zang 2013	939	10480	0.09	[0.08; 0.10]
Evans 2007	1742	13897	0.13	[0.12; 0.13]
Cho 2015	166	28392	0.01	[0.00; 0.01]
Random effects model		64559	0.07	[0.03; 0.13]
Prediction interval				[0.00; 0.57]
Heterogeneity: χ^2_{8} = 2069.89 (P < .001), I^2 = 100%				
**data type = administrative database**				
Park 2015	1810	18779	0.10	[0.09; 0.10]
Court 2014	5348	291169	0.02	[0.02; 0.02]
Hamedani 2019	472871	47582342	0.01	[0.01; 0.01]
Random effects model		47892290	0.03	[0.01; 0.07]
Prediction interval				[0.00; 1.00]
Heterogeneity: χ^2_{2} = 11064.06 (P < .001), I^2 = 100%				
Random effects model		48568279	0.10	[0.07; 0.16]
Prediction interval				[0.01; 0.64]
Heterogeneity: χ^2_{27} = 549365.68 (P < .001), I^2 = 100%				

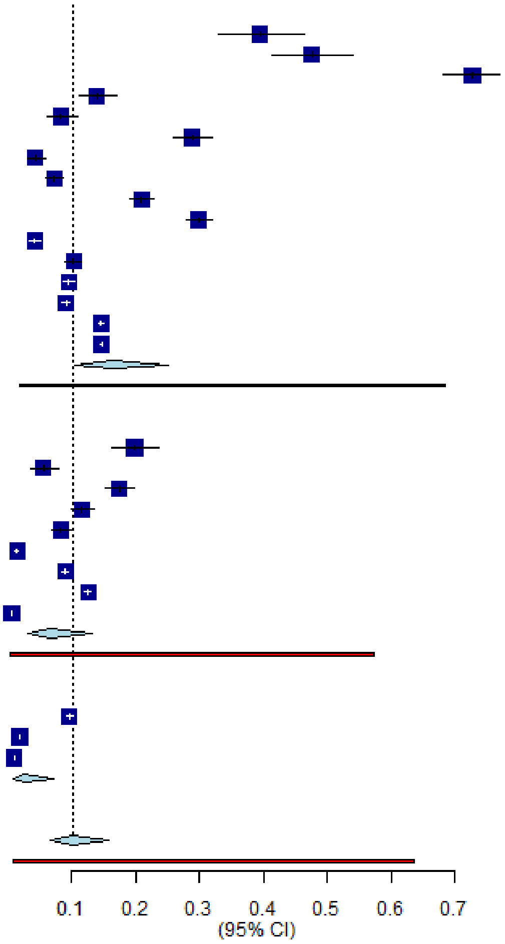

Supplementary Materials S7 - Forest plot of frequency of depression in visual impairment subjects

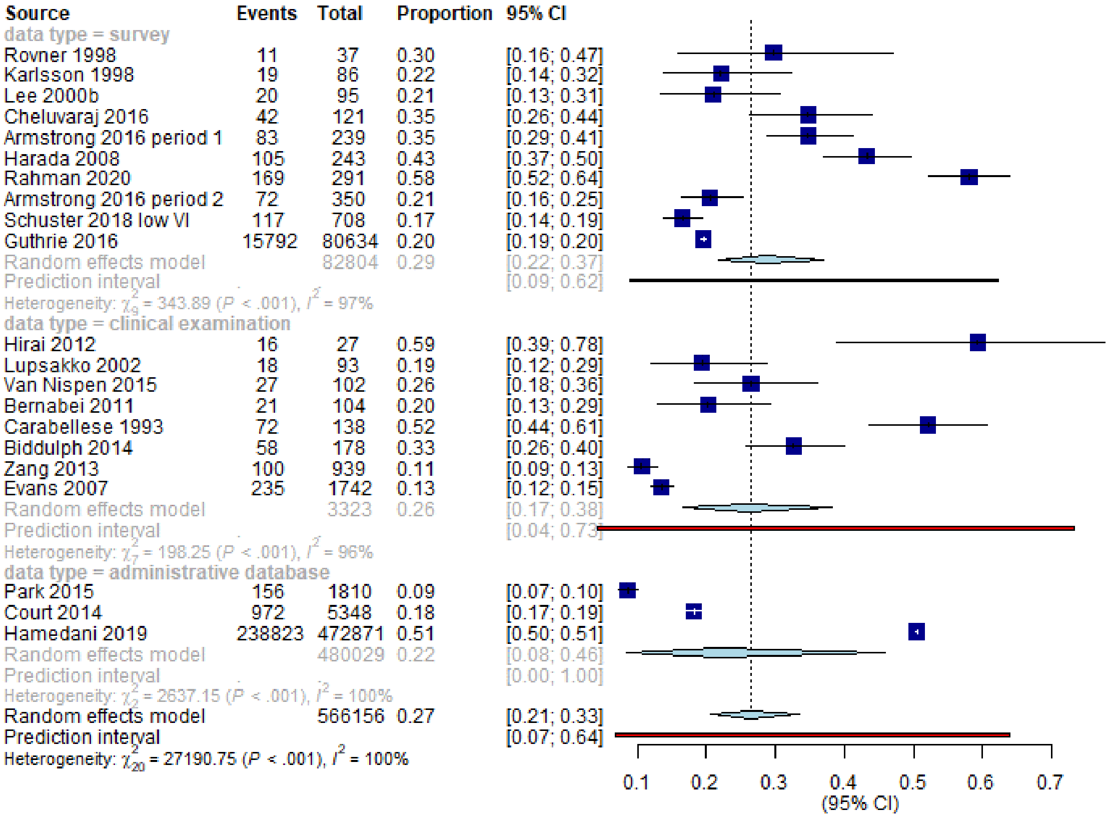

Supplementary Materials S8 - Forest plot of frequency of visual impairment in depressed subjects

Source	Events	Total	Proportion	95% CI
data type = survey				
Karlsson 1998	19	44	0.43	[0.28; 0.59]
Cheluvaraj 2016	42	73	0.58	[0.45; 0.69]
Rovner 1998	11	82	0.13	[0.07; 0.23]
Rahman 2020	169	222	0.76	[0.70; 0.82]
Harada 2008	105	249	0.42	[0.36; 0.49]
Lee 2000b	20	345	0.06	[0.04; 0.09]
Armstrong 2016 period 2	72	395	0.18	[0.15; 0.22]
Armstrong 2016 period 1	83	416	0.20	[0.16; 0.24]
Schuster 2018 low VI	117	552	0.21	[0.18; 0.25]
Guthrie 2016	15792	87519	0.18	[0.18; 0.18]
Random effects model		89897	0.28	[0.16; 0.43]
Prediction interval				[0.03; 0.84]
Heterogeneity: $\chi^2_9 = 483.47$ ($P < .001$), $I^2 = 98\%$				
data type = clinical examination				
Lupsakko 2002	18	72	0.25	[0.16; 0.37]
Biddulph 2014	58	146	0.40	[0.32; 0.48]
Hirai 2012	16	183	0.09	[0.05; 0.14]
Van Nispen 2015	27	183	0.15	[0.10; 0.21]
Carabellese 1993	72	389	0.19	[0.15; 0.23]
Bernabei 2011	21	688	0.03	[0.02; 0.05]
Zang 2013	100	749	0.13	[0.11; 0.16]
Evans 2007	235	1049	0.22	[0.20; 0.25]
Random effects model		3459	0.15	[0.09; 0.25]
Prediction interval				[0.02; 0.61]
Heterogeneity: $\chi^2_7 = 157.18$ ($P < .001$), $I^2 = 96\%$				
data type = administrative database				
Park 2015	156	653	0.24	[0.21; 0.27]
Court 2014	972	36039	0.03	[0.03; 0.03]
Random effects model		36692	0.08	[0.02; 0.33]
Prediction interval				
Heterogeneity: $\chi^2_1 = 621.33$ ($P < .001$), $I^2 = 100\%$				
Random effects model		130048	0.20	[0.13; 0.29]
Prediction interval				[0.02; 0.73]
Heterogeneity: $\chi^2_{19} = 4529.63$ ($P < .001$), $I^2 = 100\%$				

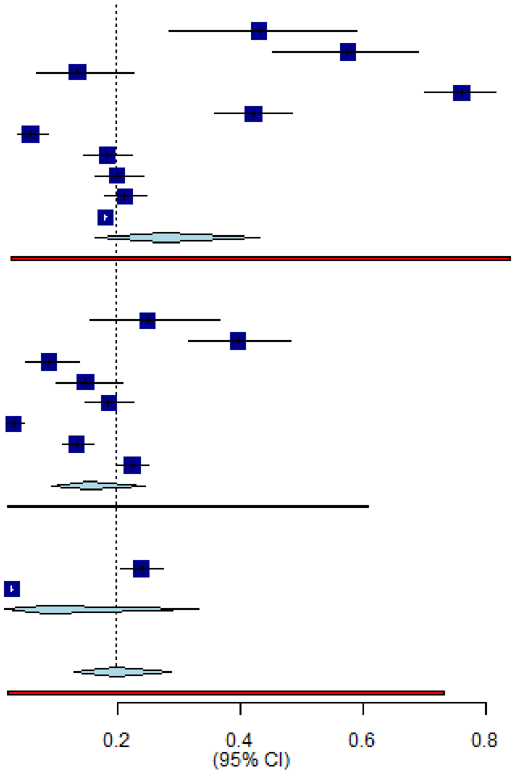

Supplementary Materials S9 - Forest plot of unadjusted association between visual impairment and depression

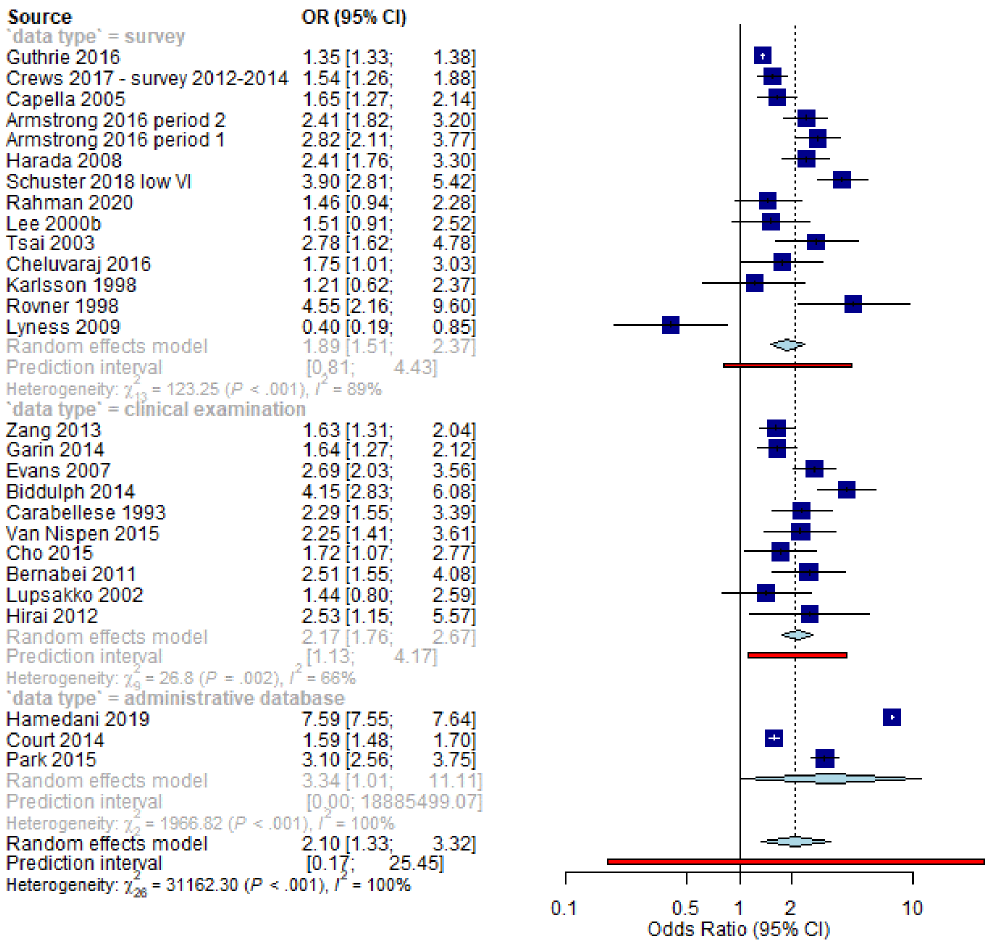

Supplementary Materials S10 - Forest plot of adjusted association between vision impairment and depression

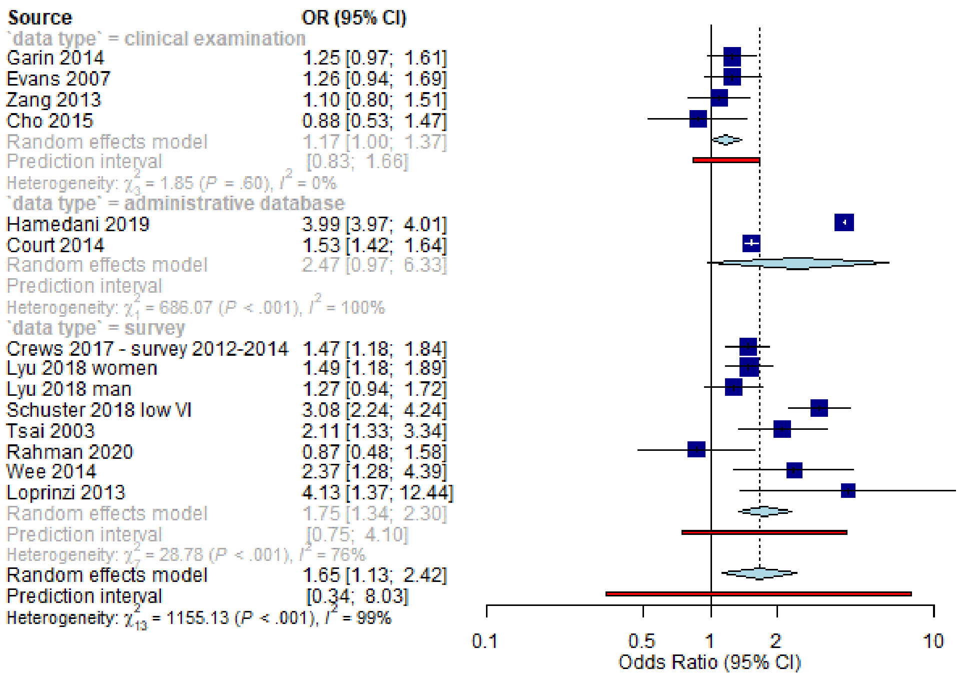

Supplementary Materials S11 - Forest plot of unadjusted association between VI and depression by depression diagnostic tool

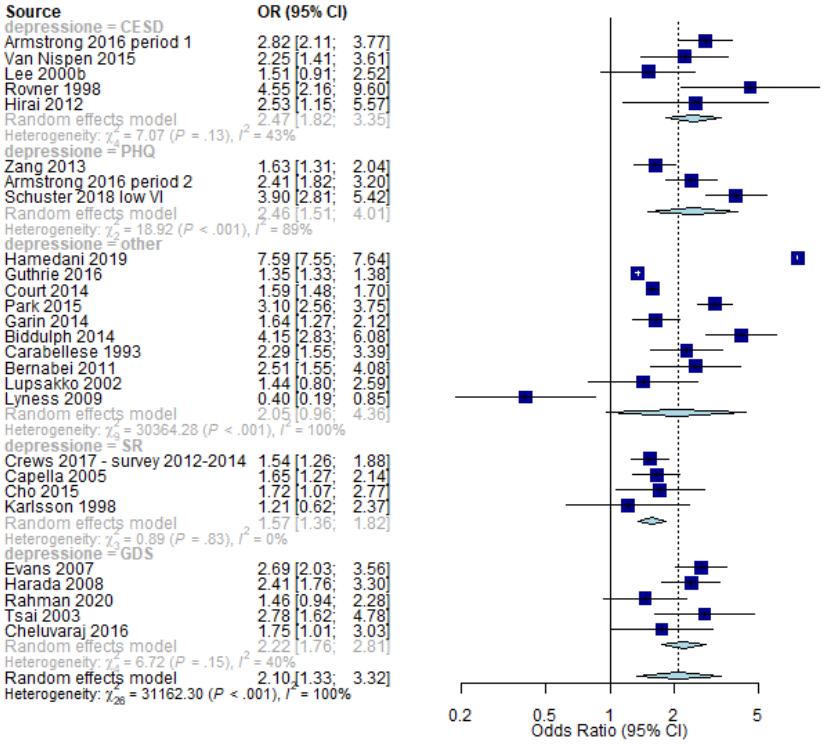

Supplementary Materials S12 - Forest plot of unadjusted association between VI and depression by age inclusion criteria

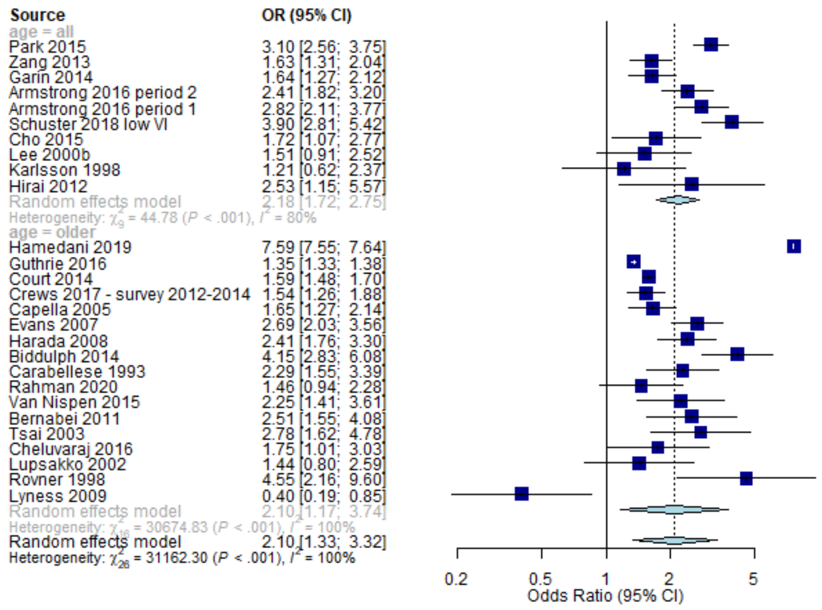

Supplement: Supplementary file 1 [file jcm-11-02412-s001.zip › jcm-1638656-supplementary.pdf]
